# Overall Survival of Patients With Pyruvate Kinase Deficiency in the UK: A Real‐World Study

**DOI:** 10.1002/jha2.70009

**Published:** 2025-03-03

**Authors:** Patrick Foy, Sara Higa, Jing Zhao, Karabo Keapoletswe, Lorena Cirneanu, Alessandra Venerus, Louise Raiteri, Erik Landfeldt, Eleonora Iob, Louise Lombard, Junlong Li, Erin Zagadailov

**Affiliations:** ^1^ Division of Hematology and Oncology Medical College of Wisconsin Milwaukee Wisconsin USA; ^2^ Agios Pharmaceuticals, Inc. Cambridge Massachusetts USA; ^3^ IQVIA Limited London UK; ^4^ IQVIA Solutions Italy Srl Milan Italy; ^5^ IQVIA Stockholm Sweden; ^6^ Institute of Epidemiology and Healthcare University College London London UK

**Keywords:** overall survival, PK deficiency, real‐world study

1

To the Editor,

Pyruvate kinase (PK) deficiency is a rare, congenital haemolytic anaemia, with an estimated diagnosed prevalence in Western populations of between 3.2 and 8.5 per million [1]. Patients with PK deficiency have a spectrum of significant, long‐term complications that include iron overload, liver disease, osteopenia/bone fragility, biliary events and pulmonary hypertension [1, 2, 3, 4]. It has been reported that some of these clinical complications, including iron overload and pulmonary hypertension, are associated with a lower health‐related quality of life in patients with PK deficiency [5]. The overlap in clinical and haematological features of PK deficiency with other hereditary anaemias, as well as its clinical heterogeneity, often hinders diagnosis of the disease [6]. Due to the rarity of PK deficiency and its common misdiagnosis [6], the current understanding of the disease burden and the impact of PK deficiency on patient survival is limited. A better understanding of survival outcomes could improve disease management, timing of treatment intervention and healthcare resource utilisation.

To better characterise the survival outcomes of these patients, this retrospective cohort study evaluated overall survival (OS) of patients with PK deficiency and matched non‐PK deficiency controls in the United Kingdom (UK), using data from the Clinical Practice Research Datalink (CPRD). The CPRD is comprised of two longitudinal primary care databases, CPRD Aurum and CPRD GOLD, which cover 20% and 4% of the UK population, respectively. These databases were linked to secondary care databases, Hospital Episode Statistics and the Office of National Statistics [7].

Cases with PK deficiency included males and females of any age with at least one coded clinical term within the CPRD Aurum/GOLD databases (MedCode) for PK deficiency at any time in their medical history (Table S1). Each case was matched with five control subjects based on year of birth, sex, database listing (e.g., a patient from CPRD Aurum was matched with a control from CPRD Aurum), date of first appearance of a PK deficiency diagnosis code and registered general practice. Controls were defined (using MedCodes) as those who had no PK deficiency or other acquired or congenital anaemias and with at least one medical record of any type in the same calendar year as the first observed occurrence of a PK deficiency diagnosis code for their matched case.

For cases, the index date was defined as the date of the first observed occurrence of a PK deficiency diagnosis code. For controls, the index date was defined as the first available medical record within the same calendar year as the index date for their matched case. Clinical data, such as complications, were reported from the earliest available date in patients’ records. Patient demographics and clinical characteristics at the index date were summarised descriptively.

Survival outcomes, including OS from birth and OS post‐index, were evaluated. OS from birth was defined as time from birth to death; patients whose death was not observed during the study period were censored at the study end date (31 October 2020). OS post‐index was defined as the time from the index date to the occurrence of the same criteria above. OS from birth and OS post‐index were estimated using the Kaplan–Meier method. Hazard ratios (HRs) between cases and controls were estimated from univariable Cox models using cohort (PK deficiency vs. control) as the only covariate. The proportional hazards assumption was checked based on the Schoenfeld residual plots.

A total of 89 cases were identified and matched with 445 controls. Both cohorts were 44% female (Table S2). Mean age (standard deviation) at index was similar between cases (24.7 [21.4] years) and controls (24.5 [21.3] years). Median follow‐up from birth was 41.3 years (quartile [Q]1–Q3: 26.3–56.3) for both PK deficiency and controls; median follow‐ups from index were 16.7 years (Q1–Q3: 8.7–22.8) and 16.8 years (Q1–Q3: 8.8–23.0), respectively (Table S2). Mean (standard deviation) haemoglobin level, at the latest available haemoglobin measurement during follow‐up, was 11.0 g/dL (2.2) in patients with PK deficiency and 14.1 g/dL (1.5) in matched controls (Table S2). A total of 59 (66.3%) patients with PK deficiency and 10 (2.2%) matched controls had a record of folic acid prescription between the index date and the end of follow‐up (Table S2). A greater proportion of patients with PK deficiency had the following complications compared with matched controls, including biliary events (38.2% vs. 3.2%), spleen disorders (24.7% vs. < 1.1%) and cardiac complications (20.2% vs. 7.9%; Table S3).

During follow‐up, eight deaths were observed among patients with PK deficiency, and nine in the matched control cohort; median ages at death were 53.9 years (range: 29.3–76.9) and 64.1 years (range: 52.9–79.3), respectively.

From birth, OS was significantly shorter for patients with PK deficiency, with five times the risk of death compared with matched controls (HR 5.0 [95% confidence interval (CI): 1.9–13.4; *p* = 0.0012]; Figure 1A; Table 1). Median OS from birth was 76.9 years for patients with PK deficiency and was not reached in the matched control cohort. At 55 years of age, the estimated probability of survival was 92% (95% CI: 84%–100%) among patients with PK deficiency and 99% (95% CI: 97%–100%) among controls. Corresponding estimates at 65 years of age were 86% (95% CI: 75%–100%) and 95% (95% CI: 90%–100%), respectively.

**FIGURE 1:**
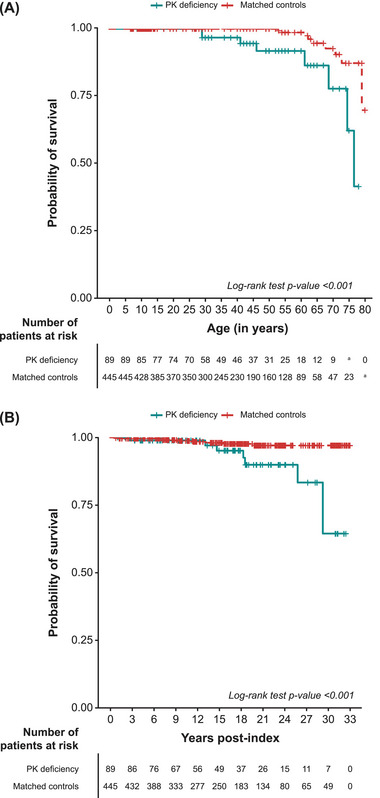
(A) OS from birth and (B) OS post‐index for patients with PK deficiency and matched controls. Abbreviations: CPRD, Clinical Practice Research Datalink; OS, overall survival; PK, pyruvate kinase. Masking rule: number of patients < 5. Index for patients with PK deficiency was the first occurrence of a PK deficiency diagnosis code, which could appear at any time in the patient's records, either before or after CPRD registration. Index for matched controls was the first medical record in the same calendar year as the index date for the patient with PK deficiency.

**TABLE 1:** HR of overall survival in patients with PK deficiency compared with matched controls.

	Number of patients	Number of deaths	HR (95% CI)	*p* value
From birth				
PK deficiency	89	8	5.0 (1.9–13.4)	0.0012
Matched controls	445	9		
Post‐index				
PK deficiency	89	8	4.6 (1.8–11.9)	0.0018
Matched controls	445	9		

*Note*: The index for patients with PK deficiency was the first occurrence of a PK deficiency diagnosis code, which could appear at any time in the patient's records, either before or after CPRD registration. Index for matched controls was the first medical record in the same calendar year as the index date for the patient with PK deficiency. Each case was matched with five control subjects based on year of birth, sex, database listing (e.g., a patient from CPRD Aurum was matched with a control from CPRD Aurum), date of first appearance of a PK deficiency diagnosis code and registered general practice. The HR and corresponding *p* value were estimated from a univariable Cox model.

Abbreviations: CI, confidence interval; CPRD, Clinical Practice Research Datalink; HR, hazard ratio; PK, pyruvate kinase.

OS post‐index was significantly lower in the PK deficiency cohort compared with matched controls (HR 4.6 [95% CI: 1.8–11.9; *p* = 0.0018]; Table 1). The Schoenfeld residue plot may suggest a non‐random pattern against time, therefore the HR should be interpreted with caution. The median OS from the index was not reached in either cohort. The estimated probability of survival at 15 years post‐index was 95% (95% CI: 90%–100%) among patients with PK deficiency and 98% (95% CI: 97%–100%) among controls. The estimated probability of survival was markedly decreased for patients with PK deficiency 30 years post‐index (65% [95% CI: 44%–96%]) compared with controls (97% [95% CI: 95%–99%]; Figure 1B).

This is the first study to evaluate OS of patients with PK deficiency in the UK. Compared with matched controls, patients with PK deficiency were found to have significantly lower OS, with 4.6 to 5 times the risk of death from both index and birth. The results add to a growing body of evidence that PK deficiency is associated with reduced survival, also including a recent study of patients from the US Veterans Health Administration [8]. However, our study included a larger number of patients with PK deficiency than the US study (89 vs. 18, respectively) and was conducted in a more balanced population with respect to gender (56% vs. 94% male) and the population was younger at index (approximately 25 vs. 57 years); our study was also representative of UK clinical practice compared with US veterans. Limitations of the current study include the potential for survivor bias and inaccurate disease diagnosis (e.g., due to limited availability of data in the CPRD regarding genetic testing, medical and treatment history [treatments include only those prescribed by a general practitioner, and not ‘over‐the‐counter’ medications]), low patient numbers (meaning sub‐analyses, such as outcomes per age categories could not be performed) and unknown heterozygous carriers and varying disease severity. In addition, there was potential for confounding bias during the matching process, as matching was only done on a limited set of observed variables (primarily age and sex). Further evidence based on long‐term data is needed to evaluate the life expectancy of patients with various forms of PK deficiency, according to genetic characteristics.

Our findings provide novel insights into the substantial disease burden and prevailing unmet medical needs, which highlight the importance of early, accurate diagnosis, and early intervention with appropriate therapy for patients with PK deficiency.

## Author Contributions

Sara Higa, Karabo Keapoletswe, Lorena Cirneanu, Alessandra Venerus, Louise Raiteri, Erik Landfeldt and Eleonora Iob contributed to the conception and design of the study. Patrick Foy, Sara Higa, Jing Zhao, Karabo Keapoletswe, Lorena Cirneanu, Alessandra Venerus, Louise Raiteri, Erik Landfeldt, Eleonora Iob, Louise Lombard, Junlong Li and Erin Zagadailov contributed to the interpretation of the study results, helped to draft and revise the manuscript and read and approved the final manuscript. Patrick Foy is responsible for the overall content as guarantor.

## Ethics Statement

The protocol and informed consent form were approved by an Institutional Review Board/Independent Ethics Committee at each study site and the study is being performed in accordance with the ethical principles of the Declaration of Helsinki.

## Consent

Written informed consent and assent, when appropriate, have been obtained from all enrolled patients and/or their guardians.

## Conflicts of Interest

Patrick Foy: consultancy for Alexion, Pharmacosmos and Rigel. Sara Higa: former employee and shareholder of Agios Pharmaceuticals, Inc. Jing Zhao: current employee and shareholder of Agios Pharmaceuticals, Inc. Karabo Keapoletswe: current employee of IQVIA Ltd. Lorena Cirneanu: current employee of IQVIA Ltd. Alessandra Venerus: current employee of IQVIA Solutions Italy Srl. Louise Raiteri: current employee of IQVIA Ltd. Erik Landfeldt: current employee of IQVIA Solutions Sweden AB. Eleonora Iob: current employee of IQVIA Ltd. Louise Lombard: current employee and shareholder of Agios Pharmaceuticals, Inc. Junlong Li: current employee and shareholder of Agios Pharmaceuticals, Inc. Erin Zagadailov: former employee and shareholder of Agios Pharmaceuticals, Inc.

## Supporting information



Supporting Information

## Data Availability

Qualified researchers may request access to related clinical study documents. Please send your data sharing requests to datasharing@agios.com. The following considerations will be taken into account as part of the review: 1. Language used in data and requested documents (e.g., English, or other). 2. Informed consent language with respect to allowance for data sharing. 3. Potential conflict of interest or competitive risk.
