# Supplementary material for: Overall Survival of Patients With Pyruvate Kinase Deficiency in the UK: A Real‐World Study
Source: EJHaem. 2025 Mar 3;6(2):e70009. doi: 10.1002/jha2.70009 (PMC11875059; doi:10.1002/jha2.70009)
Supplement: Supplementary file 1 — Supporting Information [file JHA2-6-e70009-s001.docx]

Supplementary material

Overall survival of patients with pyruvate kinase deficiency in the UK: A real-world study

Patrick Foy, Sara Higa, Jing Zhao, Karabo Keapoletswe, Lorena Cirneanu, Alessandra Venerus, Louise Raiteri, Erik Landfeldt, Eleonora Iob, Louise Lombard, Junlong Li, Erin Zagadailov

| TABLE S1 CPRD MedCodes used to identify patients with PK deficiency. | |
| --- | --- |
| **CPRD Aurum database** | **Description** |
| 4363551000006119 | Deficiency of pyruvate kinase |
| 294000010 | Haemolytic anaemia due to pyruvate kinase deficiency |
| 4761371000006119 | Haemolytic anaemia due to pyruvate kinase deficiency |
| 4363581000006110 | PK – Pyruvate kinase deficiency |
| 4363591000006113 | Pyruvate kinase deficiency |
| 3714051000006113 | Pyruvate kinase deficiency anaemia |
| 3714071000006115 | Pyruvate kinase deficiency anaemia |
| 3714041000006111 | HNSHA due to pyruvate kinase deficiency |
| 3714111000006111 | Hereditary nonspherocytic haemolytic anaemia due to pyruvate kinase deficiency |
| 3714091000006119 | Hereditary nonspherocytic haemolytic anaemia due to pyruvate kinase deficiency |
| 3714101000006113 | Hereditary nonspherocytic haemolytic anaemia due to pyruvate kinase deficiency |
| 3714061000006110 | PK deficiency anaemia |
| 3714081000006117 | PK deficiency anaemia |
| **CPRD GOLD database** | **Description** |
| 55561 | Haemolytic anaemia due to pyruvate kinase deficiency |

Abbreviations: CPRD, Clinical Practice Research Datalink; HNSHA, Hereditary nonspherocytic haemolytic anaemia; MedCode, coded clinical term within the CPRD Aurum/GOLD databases; PK, pyruvate kinase.

| **TABLE S2** Demographics and follow-up for patients with PK deficiency and matched controls. | | |
| --- | --- | --- |
| **Variables** | **PK deficiency cohort**  **(*N* = 89)** | **Matched non-PK deficiency cohort**  **(*N* = 445)** |
| Demographics |  |  |
| Age at index (years), mean (SD) | 24.7 (21.4) | 24.5 (21.3) |
| Sex, *n* (%) |  |  |
| Male | 50 (56.2) | 250 (56.2) |
| Female | 39 (43.8) | 195 (43.8) |
| Follow-up period |  |  |
| From birth (years), median (Q1–Q3) | 41.3 (26.3–56.3) | 41.3 (26.3–56.3) |
| From index (years), median (Q1–Q3) | 16.7 (8.7–22.8) | 16.8 (8.8–23.0) |
| Laboratory values at latest measurement during follow-up |  |  |
| Haemoglobin^a^ (g/dL), mean (SD) | *N* = 69  11.0 (2.2) | *N* = 252  14.1 (1.5) |
| Total bilirubin (µmol/L), mean (SD) | *N* = 44  51.8 (42.9) | *N* = 171  10.4 (5.1) |
| Reticulocyte percentage (%), mean (SD) | *N* = 13  12.9 (18.5) | *N* = 0  NA |
| Ferritin (µg/L), mean (SD) | *N* = 38  343.7 (279.6) | *N* = 100  100.4 (107.7) |
| Folic acid prescription^b^, *n* (%) | 59 (66.3) | 10 (2.2) |
| Other characteristics, *n* (%) |  |  |
| Database |  |  |
| CPRD Aurum | 81 (91.0) | 405 (91.0) |
| CPRD GOLD | 8 (9.0) | 40 (9.0) |
| Year of PK deficiency diagnosis |  |  |
| <1990–1995 | 15 (16.9) | 75 (16.9) |
| 1996–2000 | 17 (19.1) | 85 (19.1) |
| 2001–2005 | 19 (21.3) | 95 (21.3) |
| 2006–2010 | 12 (13.5) | 60 (13.5) |
| 2011–2015 | 17 (19.1) | 85 (19.1) |
| 2016–2020 | 9 (10.1) | 45 (10.1) |

A masking rule was applied for variables with a frequency count of <5 and percentages were not calculated.

Abbreviations: CPRD, Clinical Practice Research Datalink; NA, not available; PK, pyruvate kinase; Q1, first quartile; Q3, third quartile; RBC, red blood cell; SD, standard deviation.

^a^Haemoglobin test values within 61 days after an RBC transfusion were excluded from the analysis.

^b^Assessed from index date to the end of follow-up.

| TABLE S3 Lifetime history of clinical complications of patients with PK deficiency and matched controls. | | | | |
| --- | --- | --- | --- | --- |
|  | **PK deficiency**  **(*N* = 89)** | | **Matched controls**  **(*N* = 445)** | |
| Complication | *n* | % | *n* | % |
| Liver failure | <5 | NC | 0 | 0.0 |
| Other liver complications^a^ | 10 | 11.2 | <5 | NC |
| Cardiac complications^b^ | 18 | 20.2 | 35 | 7.9 |
| Iron overload^c^ | 15 | 16.9 | <5 | NC |
| Biliary events^d^ | 34 | 38.2 | 14 | 3.1 |
| Spleen disorders^e^ | 22 | 24.7 | <5 | NC |
| Bone health problems^f^ | 17 | 19.1 | 80 | 18.0 |
| General infections^g^ | 12 | 13.5 | 8 | 1.8 |
| Transfusion-related reactions^h^ | 7 | 7.9 | 0 | 0.0 |
| Infections with encapsulated bacteria^i^ | 5 | 5.6 | 5 | 1.1 |
| Pulmonary hypertension | <5 | NC | <5 | NC |
| Thromboembolic events^j^ | <5 | NC | <5 | NC |
| Endocrine complications^k^ | 9 | 10.1 | 31 | 7.0 |
| Anxiety or depression | 26 | 29.2 | 155 | 34.8 |
| Skin ulceration | <5 | NC | <5 | NC |
| Lower limb ulcer | <5 | NC | <5 | NC |

Complications are reported from the earliest available data and could appear at any time in the patient’s records, either before or after CPRD registration. A masking rule was applied for complications with a frequency count of <5 and percentages were not calculated.

Abbreviations: CPRD, Clinical Practice Research Datalink; MedCode, coded clinical term within the CPRD Aurum/GOLD databases; NC, not calculated; PK, pyruvate kinase.

^a^Other liver complications includes: cirrhosis, hepatomegaly and non-alcoholic fatty liver.

^b^Cardiac complications includes: arrythmia, congestive heart failure and left ventricular hypertrophy.

^c^Iron overload was defined as ferritin >1000 μg/L, presence of a MedCode for iron chelation or presence of any MedCode that stated ‘iron overload’.

^d^Biliary events includes: bile duct stone, cholangitis, cholecystitis and gallstones.

^e^Spleen disorders includes: accessory spleen, hypersplenism, spleen rupture, splenic haematopoiesis, splenomegaly and splenomegaly neonatal.

^f^Bone health problems includes: bone pain, fragility fracture, osteopenia and osteoporosis.

^g^General infections includes: hepatitis B, hepatitis C and sepsis.

^h^Transfusion-related reactions includes: alloimmunisation, autoimmune haemolytic anaemia, febrile non-haemolytic transfusion reaction, transfusion-associated dyspnoea and transfusion-related acute lung injury.

^i^Infections with encapsulated bacteria includes: *Escherichia* infection, *Haemophilus* infection, pneumonia streptococcal and *Pseudomonas* infection.

^j^Thromboembolic events includes: deep vein thrombosis and pulmonary embolism.

^k^Endocrine complications includes: Basedow’s disease, diabetes mellitus, growth hormone deficiency, Hashimoto’s disease, hyperthyroidism, hypoparathyroidism, hypothyroidism, secondary hypogonadism and thyroid mass.
